# Enabling free‐breathing background suppressed renal pCASL using fat imaging and retrospective motion correction

**DOI:** 10.1002/mrm.27723

**Published:** 2019-03-18

**Authors:** Isabell K. Bones, Anita A. Harteveld, Suzanne L. Franklin, Matthias J. P. van Osch, Jeroen Hendrikse, Chrit T. W. Moonen, Clemens Bos, Marijn van Stralen

**Affiliations:** ^1^ Center for Image Sciences University Medical Center Utrecht Utrecht the Netherlands; ^2^ C. J. Gorter Center for High Field MRI, Department of Radiology Leiden University Medical Center Leiden the Netherlands; ^3^ Department of Radiology University Medical Center Utrecht Utrecht the Netherlands

**Keywords:** background suppression, fat‐navigator, motion artifacts, pCASL, registration, renal perfusion

## Abstract

**Purpose:**

For free‐breathing renal perfusion imaging using arterial spin labeling (ASL), retrospective image realignment has been found essential to reduce subtraction artifacts and, independently, background suppression has been demonstrated to reduce physiologic noise. However, negative results on ASL precision and accuracy have been reported for the combination of both. In this study, the effect of background suppression ‐level in combination with image registration on free‐breathing renal ASL signal quality, with registration either on ASL‐images themselves or guided by additionally acquired fat‐images, was investigated. The results from free‐breathing acquisitions were compared with the reference paced‐breathing motion compensation strategy.

**Methods:**

Pseudocontinuous ASL (pCASL) data with additional fat‐images were acquired from 10 subjects at 1.5T with varying background suppression levels during free‐breathing and paced‐breathing. Images were registered using the ASL‐images themselves (ASLReg) or using their corresponding fat‐images (FatReg). Temporal signal‐to‐noise ratio (tSNR) served to evaluate precision and perfusion weighted signal (PWS) to assess accuracy.

**Results:**

In combination with image registration, background suppression significantly improved tSNR by 50% (*P *< .05). For heavy suppression, ASLReg and FatReg showed similar performance in terms of tSNR and PWS. Background suppression with two inversion pulses induced a small, nonsignificant (*P *> .05) PWS reduction, but increased PWS accuracy. When applying heavy background suppression, free‐breathing acquisitions resulted in similar ASL‐quality to paced‐breathing acquisitions.

**Conclusion:**

Background suppression was found beneficial for free‐breathing renal pCASL precision without compromising accuracy, despite motion challenges. In combination with ASLReg or FatReg, background suppression enabled clinically viable free‐breathing renal pCASL.

## 1 INTRODUCTION

For patients with diseased kidneys, e.g. chronic kidney disease, and kidney transplant recipients, it is vital to assess renal function for monitoring of the disease or transplant status. In current clinical practice, blood and urine tests are used to assess renal function by monitoring waste and fluid removal from the blood or by measuring protein content in the urine. A disadvantage of those tests is that they are not sensitive to poor single kidney or regional kidney dysfunction. Alternatively, a kidney biopsy can be performed. However, this procedure is highly invasive and offers merely selective local information and thus suffers from sampling errors.^1^

Different imaging techniques have been proposed to measure renal perfusion, mostly utilizing nuclear tracers (PET‐MRI) or contrast agents [dynamic contrast‐enhanced (DCE)‐MRI].^2^, ^3^ However, these techniques are invasive and require intravenous contrast or tracer injection, which are potentially nephrotoxic or have nephrogenic toxicity. Therefore, they are generally considered less safe or suitable in patients with kidney damage or in the pediatric population. In the past years, renal perfusion measurement using ASL‐MRI has raised research interest and has shown promising results in providing consistent perfusion measurements.^3^, ^4^ The ASL‐MRI is based upon the magnetic labeling of blood, which will subsequently act as an endogenous tracer. This makes ASL a repeatable and noninvasive method. To increase SNR and reduce the influence of noise and artifacts in the resulting PWS, multiple repetitions of the same ASL measurement are acquired and averaged. Since ASL is a subtraction technique, it is very sensitive to motion. This makes renal ASL challenging in the presence of respiratory and peristaltic motion. Motion can be present between images of repeated acquisitions as well as between images of the same label–control pair. As a consequence, structural mismatch between images results in subtraction artifacts that could potentially perturb the perfusion signal.

Several strategies have been proposed to minimize the effect of motion. Respiratory triggering based on the monitored respiratory signal can be performed to acquire all source images in expiration.^5^, ^6^ This strategy does account for bulk respiratory motion, thereby improving ASL quality. Yet, scan time increases and a direct relation between the monitored respiratory signal and target organ position is assumed. Alternatively, breath holding and paced‐breathing during acquisition have been shown to reduce subtraction artifacts significantly.^6^, ^7^, ^8^ However, these motion compensation strategies require subject cooperation, place a constraint on total acquisition time, and may be difficult to perform successfully in certain subjects (e.g. elderly or pediatric populations). To alleviate the need for patient cooperation and reduce scan time, free‐breathing acquisitions have been suggested.^6^, ^8^, ^9^, ^10^

With free‐breathing renal ASL acquisitions, issues such as bulk motion‐induced subtraction artifacts arise. When motion in free‐breathing renal ASL series is not accounted for, perfusion maps have a blurred appearance with low SNR due to subtraction artifacts.^6^, ^8^ Retrospective registration has been found essential to reduce these subtraction artifacts and yield sharper perfusion images.^6^, ^10^ To reduce physiologic noise and subtraction errors due to motion, the application of background suppression (BGS) pulses has been proposed.^11^, ^12^, ^13^, ^14^ For brain ASL the benefits of BGS have already been widely demonstrated, resulting in the recommendation of BGS usage in the recent brain‐ASL consensus paper by Alsop et al.^14^ For kidney ASL a positive effect of BGS on visual perfusion quality and a lower measurement variance have also been reported.^5^, ^6^, ^7^, ^8^ Interestingly, negative results in terms of SNR have been reported for the combination of image registration and BGS,^6^ questioning the feasibility of image registration for BGS ASL‐images.

The lack of static tissue signal, due to BGS, challenges motion‐correction algorithms to realign the images accurately.^14^, ^15^, ^16^ So far, motion was avoided by applying paced‐breathing^7^, ^8^ or outlier rejection.^6^ We sought to assess the feasibility of image‐based registration on BGS images, while achieving full kidney coverage.

Recently so‐called fat‐navigators have been proposed to correct target images for motion.^17^, ^18^, ^19^ For example, in the brain fat‐navigators have been used for prospective motion correction.^17^ An advantage of fatty tissue is that it is unaffected by contrast agents and recovers quickly from preceding BGS pulses due to the short T1. Thus, fat‐navigators could be suitable for retrospective motion correction between images with varying soft tissue contrast, as often occurs when contrast agent is injected or BGS is applied. This has led to successful application of fat‐navigators for dynamic contrast‐enhanced MRI in the breast^18^ and kidney.^19^

The aim of this study was to investigate the feasibility of background suppressed renal ASL in combination with retrospective image registration during free‐breathing acquisition. To this end, the effect of BGS level on precision and accuracy of renal ASL perfusion measurement was evaluated. Moreover, we introduced the acquisition of additional fat‐images in a pCASL sequence and tested their feasibility for image registration guidance compared with conventional guidance by the ASL‐images themselves. Finally, results from free‐breathing acquisitions were compared with the reference paced‐breathing motion compensation strategy.

## 2 METHODS

This study was performed with approval from the institutional review board, and written informed consent was obtained from all subjects.

The MR experiment consisted of three parts that were acquired consecutively in one session for each subject. In part 1, settings for the acquisition of additional fat‐images for image registration guidance (referred to as FatReg), as previously explored,^20^ were systematically varied to study its performance. In part 2, the effect of various BGS levels, with subsequent image registration, on free‐breathing renal ASL was evaluated. The performance of motion correction of BGS ASL using either the ASL‐images themselves, image‐based registration based on ASL‐images (ASLReg), or our proposed FatReg technique was investigated as well. Finally, in part 3, free‐breathing acquisition was compared with the paced‐breathing strategy, as a reference.

### 2.1 Image acquisition

#### 2.1.1 Imaging sequence

Arterial spin labeling was performed using balanced pCASL consisting of a train of short, repeated RF pulses (Hann‐shaped, 480‐μs duration, 1210‐μs pulse spacing, average *B*1 = 1.5 μT) in combination with a switching slice‐selective gradient (average strength 0.36 mT^‐m^), as implemented by Philips. Labeling was applied in the aorta above the kidneys for a duration of 1500 ms, followed by a postlabeling delay of 1500 ms allowing for inflow of the label into the tissue of the kidneys. Ten repetitions, each consisting of one label‐control pair, were acquired for signal averaging. A single‐shot gradient echo planar imaging 2D multislice readout was used with an 80 × 81 acquisition matrix and an echo planar imaging factor of 55, parallel imaging factor 1.5, and phase‐encoding bandwidth of 30.9 Hz/pixel. Five coronal slices were acquired in ascending order (anterior‐posterior) with a slice gap of 1 mm, covering a 244 mm × 244 mm field of view with a reconstructed voxel size of 2.54 × 2.54 × 6 mm^3^. Phase encoding direction was feet–head, and saturation slabs superior and inferior to the imaging volume were used to suppress unwanted signal aliasing. Additionally, fat suppression was achieved by application of a spectrally selective, non‐spatially selective inversion recovery pulse (SPIR) using an angle of 120 degrees. Consequently, TR/TE were 6500/19 ms with a readout time of 62 ms per slice, giving a total scan time of 2:30 minutes; the long TR was required for paced‐breathing acquisition. *B*0 shimming covered the whole FOV, hence including the labeling slab and the aorta.

#### 2.1.2 Fat‐image acquisition

**Figure 1:**
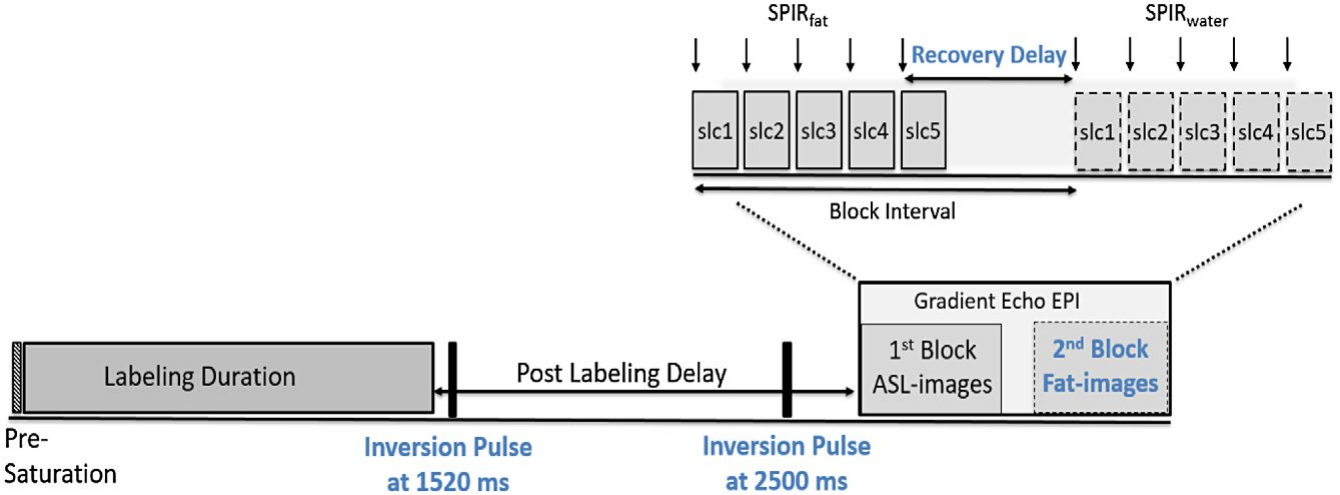
Block diagram showing the multislice pCASL, including the fat‐navigator images and background suppression (setting: BGS2M), using a multiblock gradient echoplanar imaging readout. Detailed information about the two blocks of the readout is shown in the zoomed section (top right). In the first block ASL‐images (slc1–slc5) are acquired, each preceded by a SPIR pulse for fat suppression. After the SPIR pulse of the last slice (slc 5) fat recovery begins and the chosen recovery delay starts. pCASL, pseudo continuous arterial spin labeling; ASL, arterial spin labeling; EPI, echo planar imaging; SPIR, spectral presaturation with inversion recovery

Fat‐images were acquired at the same location as the ASL slices after the ASL‐image readout (Figure 1). During the ASL‐image readout, each slice was preceded by a spectrally selective inversion pulse used for fat suppression (SPIR_fat_), while for the fat‐image readout the SPIR pulse was played out at the water frequency to achieve water suppression (SPIR_water_). The time between the last SPIR_fat_ and the first fat‐image excitation is referred to as recovery delay. During this time the fat signal recovers, but at the same time a delay between ASL and fat‐images is introduced and the total scan time is increased. Ultimately, the minimum recovery time is limited to the acquisition time of a single‐slice (Figure 1), which was 65 ms in this study. Choosing the delay time is a balance between minimizing the risk of motion between the acquisition of the two images and low fat signal, which might influence its usability for image registration. The impact of the recovery delay duration is investigated in Part 1.

#### 2.1.3 Background suppression

The BGS was applied to eliminate any residual magnetization modulation from preceding scans by applying four WET saturation pulses prior to labeling (presaturation) and to null static tissue signal at the time of imaging via properly timed hyperbolic secant inversion pulses.^21^ In this work, we focused on the inversion pulses played during the postlabeling delay. The number of pulses and their timings were varied to result in different levels of BGS, while maintaining positive static tissue signal. This last requirement arose from the use of only magnitude images in the ASL subtraction. Inversion pulse timings were carefully chosen considering T_1_ values for healthy and diseased/transplanted kidneys of 1057 to 1183 ms in the cortex and 1389 to 1573 ms in the medulla.^22^ Simulation in Matlab was used to verify whether the inversion pulse timings led to the desired BGS level. With BGS levels defined as the reduction of kidney cortex signal in percentage for slice 1, we aimed for mild and heavy BGS levels achieved by two or four inversion pulses. Timings are given with respect to the start of the labeling module, which coincides with the end of the presaturation. In total, five scans with different BGS were investigated. Two mild levels were chosen using two inversion pulses at 1520/2400 ms (BGS2m) and 1520/2500 ms (BGS2M) aiming for 70% and 80% suppression, respectively. The maximum suppression level of 90% was achieved by two inversion pulses at 1520/2600 ms (BGS2H) as well as using four inversion pulses played at 1501/2320/2752/2943 ms (BGS4H). The expected BGS levels were checked in one subject within a small region of interest (ROI) in the kidney cortex in slice 1.

### 2.2 MR experiments

Ten healthy subjects (age 22–60, three male) were scanned on a 1.5T MRI (Ingenia, Philips, Best, the Netherlands) using a 28‐element phased‐array receiver coil. First, a coil sensitivity reference scan and a scout image were acquired in end‐expiration. The image volume covered a cross section of both kidneys. Slices were angulated to be parallel to the back muscle, avoiding through‐plane motion as the kidneys slide along this muscle during respiration. The labeling slab was placed as high as possible inside the FOV to prevent spurious labeling of the kidneys due to respiratory displacement, while staying below the diaphragm to prevent susceptibility artifacts from the air/tissue interface at the lungs, as those could influence labeling efficiency.^14^ Additionally, the labeling slab was angulated in the sagittal view perpendicular to the aorta. At the start of each experiment, an equilibrium magnetization image (M0) was acquired using neither BGS nor labeling, while keeping the other sequence parameters constant.

#### 2.2.1 Part 1: Fat‐image acquisition optimization

The recovery delay (noted for slice 1) of the fat‐image acquisition was systematically varied. For this purpose, five acquisitions were taken during free‐breathing without BGS with varying recovery delays (65, 150, 250, 400, 750 ms). The TR was adapted accordingly (6500, 6585, 6685, 6835, 7185 ms) to keep the time between the ASL repetitions constant.

#### 2.2.2 Part 2: Effect of background suppression combined with image registration on ASL quality

Pseudocontinuous arterial spin labeling with additional fat‐images was acquired during free‐breathing with the five different BGS levels (NoBGS, BGS2m, BGS2M, BGS2H, BGS4H). In this part of the experiment, fat‐image recovery delay was set to the minimum of 65 ms. Subjects were asked to relax and breathe freely.

#### 2.2.3 Part 3: Breathing strategy

Finally, all scans with different BGS levels from Part 2 were also acquired during paced‐breathing. For that purpose, subjects were instructed before scanning and coached during scanning to hold their breath briefly in expiration during labeling and image readout. The TR was kept constant for both breathing strategies.

### 2.3 Image analysis

All acquired data were stored as magnitude images. Postprocessing was done offline in MeVisLab (MeVis Medical Solutions AG, Fraunhofer MEVIS, Bremen, Germany). Prior to image registration, fat‐images were corrected for water–fat shift in feet–head direction. Kidneys were manually segmented on the M0 images for each subject. These segmentations served kidney‐specific motion correction and were used as ROI for ASL quality assessments. All statistical testing was done using paired Wilcoxon signed rank tests with a significance level of 0.05.

#### 2.3.1 Image registration

**Figure 2:**
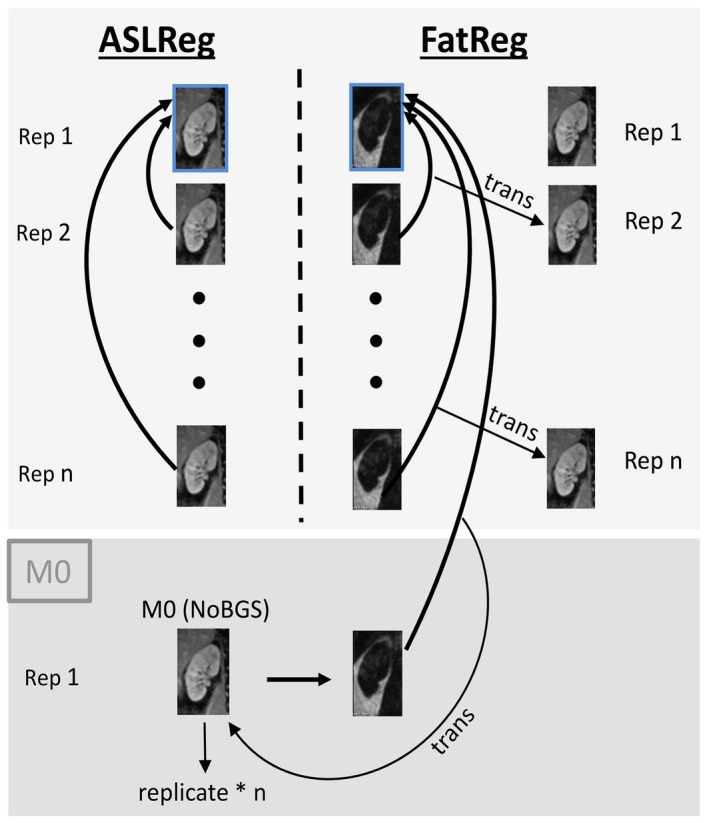
Illustration of the conventional ASLReg and the proposed FatReg. Images of all consecutive repetitions (*n*) were coregistered to the label image of the first label–control pair (fixed image, blue box). For the proposed FatReg, fat‐images guided the image registration, followed by translation of the obtained transformations to the unsubtracted ASL‐images. M0 registration was always guided by FatReg to prevent possible cross‐contrast registration failures. ASL, arterial spin labeling; ASLReg, ASL registration; FatReg, fat registraion

For all image registrations the label image of the first label–control pair served as the fixed image. Retrospective motion correction using Elastix^23^ was performed for each kidney separately by 3D registration using Euler transform and a B‐Spline Interpolator, accounting for translation only. In this study, we used the conventional approach, referred to as ASLReg, and our proposed method named FatReg (Figure 2) to correct for motion. For both approaches motion correction was applied to label and control images separately before subtraction.

- **ASLReg: **The ASL‐images themselves are used as an input for the registration algorithm.
- **FatReg:** Additionally acquired fat‐images serve as an input for the registration algorithm, followed by application of the resulting transformation to the target ASL‐images.

For motion correction, the segmented kidney ROIs were dilated by five and nine voxels in all in‐plane directions, for ASLReg and FatReg, respectively. Registration success was visually assessed and erroneous translations noted as invalid, such as through‐plane shifts or extreme translations that did not correlate to expected physiological motion, which would make the registered images unusable for perfusion analysis.

The M0 images had to be coregistered to the background suppressed ASL‐images. Initial findings indicated invalid ASLReg results for the M0 image registration depending on the BGS level. Thus, coregistration of the M0 was always performed via FatReg to prevent possible misregistration due to large image contrast differences between the M0 and the BGS ASL‐images.

#### 2.3.2 Quality parameters

After motion correction, label–control subtraction images were averaged over all repetitions (*n*) and divided by M0, yielding normalized PWIs:

(1) $$\text{PWS}\;\left[\text{\textbackslash{}\%}\right]=\frac{\frac{1}{n}\sum_{1}^{n}\;\Delta \text{M}\;}{\text{M}0}*100$$

For each scan the PWS was averaged over the entire kidney ROI in the PWIs and reported as a measure for accuracy. Extremely high or low PWS indicates subtraction artifacts or image noise. The tSNR, averaged over the entire kidney ROI in the PWIs, was calculated as the ratio of the mean voxelwise perfusion weighted signal over time (μ) and the temporal voxelwise standard deviation (*σ*):

(2) $$\text{tSNR}\;\left[\text{a}.\text{u}.\right]=\frac{\mu_{\Delta \text{M}}}{\sigma_{\Delta \text{M}}}$$

The tSNR was evaluated as a surrogate for precision of the PWS. An increase in tSNR indicates higher precision, meaning less variability in PWS between repetitions.

##### Part 1: fat‐image acquisition optimization

The FatReg performance for motion correction using fat‐images acquired with different recovery delays was assessed by tSNR and the displacement difference in the main respiratory motion direction (feet–head) between ASLReg and FatReg. tSNR is reported since motion in the label–control pairs results in subtraction artifacts, thus introducing high signal variability reflected in low tSNR. For each scan, the mean absolute displacement difference was determined by averaging over all acquired images, i.e. repetitions and label–control conditions. Since registrations were performed for left and right kidney separately, results for both kidneys were combined by averaging. In case motion is absent during the recovery delay, no displacement difference between the registration results of ASLReg and FatReg will be found.

##### Part 2: Effect of background suppression combined with image registration on ASL quality

All scans were motion corrected using the conventional ASLReg as well as FatReg, and for each registration method quality parameters (tSNR and PWS) were evaluated. For interpretation of the BGS effect on PWS a bias‐free reference value was calculated. For interpretation of the effect of BGS on PWS, a subject‐specific PWS reference value was constructed that aimed to prevent bias toward any of the BGS levels. Since we cannot assume that the PWS measured without BGS is the true perfusion, nor do we have a gold‐standard perfusion value to compare to, this reference PWS per subject was defined as the average PWS over all paced‐breathing BGS scans after conventional ASLReg (PWS_*PBref*_). The difference with the reference PWS was referred to as PWS error, which was evaluated as a function of BGS. The PWS error compounds the effects of noise and artifacts, but also systematic reduction of PWS.

##### Part 3: Breathing strategy

To assess the feasibility of free‐breathing renal pCASL, we compared the tSNR and PWS of free‐breathing and paced‐breathing scans, using either ASLReg or FatReg. For simplicity, we focused on NoBGS and the most favorable BGS level when presenting these ASL‐quality measures.

## 3 RESULTS

### 3.1 Part 1: Fat‐image acquisition optimization

**Figure 3:**
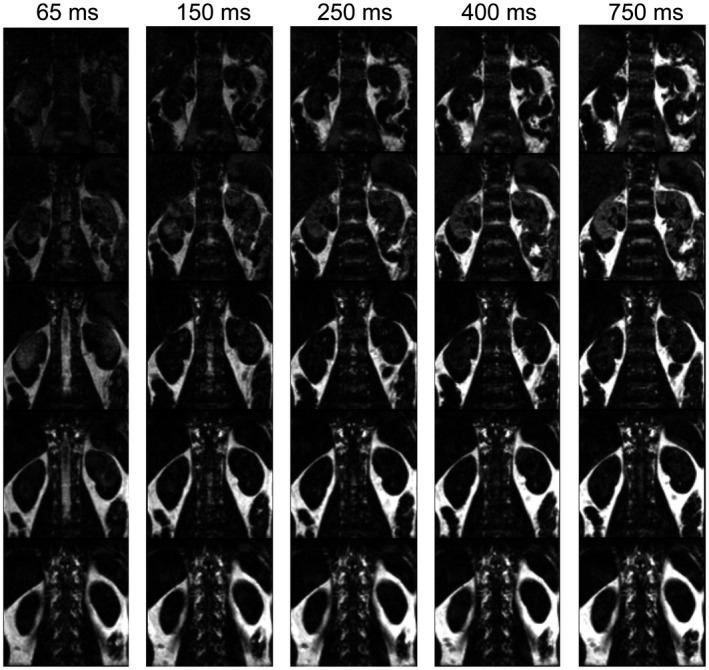
Fat‐images of five slices (rows) for one subject acquired with different recovery delays (columns). The recovery delay starts from the last excitation for the ASL‐image readout. Fat signal recovery can be seen across slices. With increasing recovery delay, fat signal increases. ASL, arterial spin labeling

**Figure 4:**
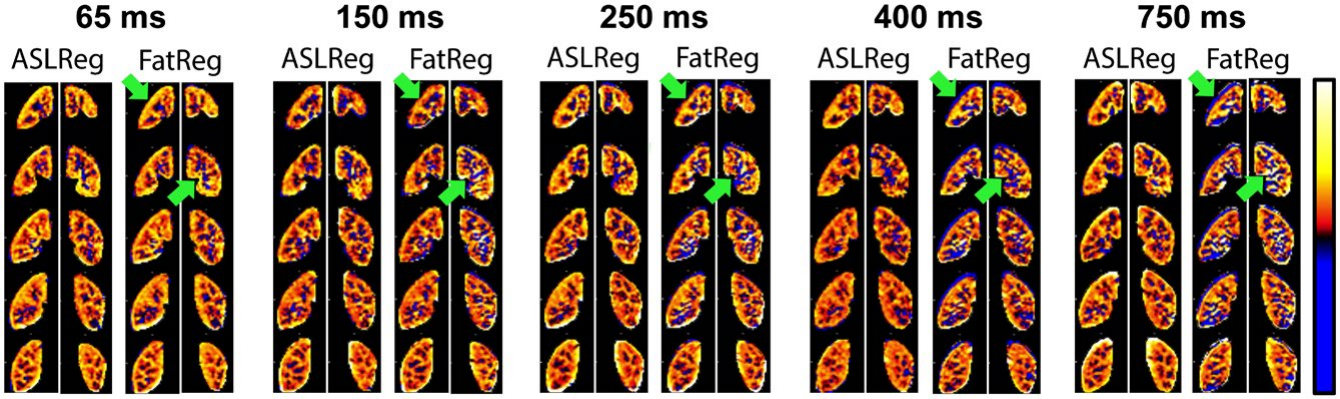
Relative perfusion‐weighted images for five slices (rows) derived after image registration on ASL‐images (ASLReg) or fat‐images (FatReg) by subtraction of label and control for all repetitions followed by averaging. Images are shown for five acquisitions with different recovery delays (columns). Residual motion between control and label images causes subtraction artifacts that appear as extreme erroneous perfusion‐weighted signal (PWS = ΔM/M0). The performance of the FatReg deteriorates with increasing delay time. Green arrows pointing at examples for signal cancellations around the right kidney and ripples of extremely high and low values in the middle of the left kidney. ASL, arterial spin labeling

**Figure 5:**
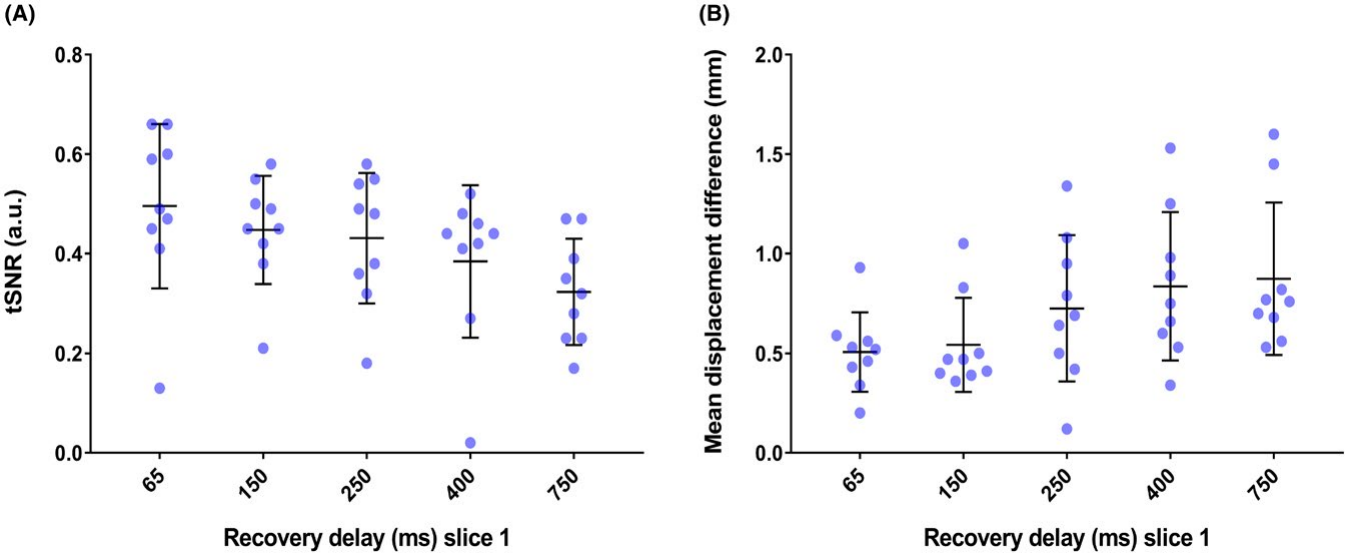
A, Temporal signal‐to‐noise ratio (tSNR) after image registration on fat‐images (FatReg) for all subjects, individually represented as blue dots. For fat‐images acquired with the shortest delay of 65 ms the highest tSNR is found; with longer delays the tSNR decreases. Outliers belong to one subject. B, Mean displacement difference of all subjects in feet–head direction between ASLReg and FatReg, determined from the translation parameters after the registration procedure. Small displacement difference values indicate agreement between motion correction based upon ASL‐images and fat‐images. ASLReg, registration guided by ASL‐images; FatReg, registration guided by fat‐images

For 1 of the 10 included subjects, time constraints required a shorter MR experiment. Thus, Part 1 was acquired for only 9 subjects. An example of fat‐images acquired with five different recovery delays is given in Figure 3, showing that overall the fat‐images visualize the kidney contours even for the shortest recovery delay of 65 ms. Along the rows of Figure 3 fat‐signal recovery across slices can be seen. Figure 4 shows PWIs of five scans acquired with different fat‐image recovery delays where motion was corrected for with ASLReg and FatReg. Visual inspection shows that with longer fat‐image recovery delays, in this case 400 ms and longer, subtraction artifacts appear when FatReg is applied (Figure 4). This is equally supported by a loss in tSNR with longer recovery delays (Figure 5A). Highest tSNR after FatReg was found using fat‐images acquired with the shortest recovery delay of 65 ms, with a small mean displacement difference of 0.5 mm between ASLReg and FatReg (Figure 5B). Longer recovery delays >400 ms significantly increased the displacement difference up to 0.8 mm.

### 3.2 Part 2: Effect of background suppression combined with image registration on ASL quality

From the 10 subjects, 9 were included in the analysis. One subject indicated difficulties with the paced‐breathing protocol, resulting in major kidney displacements with in‐plane and through‐plane motion corrupting the PWIs (Supporting Information Figure S1) and with that the calculation of the paced‐breathing reference PWS.

**Figure 6:**
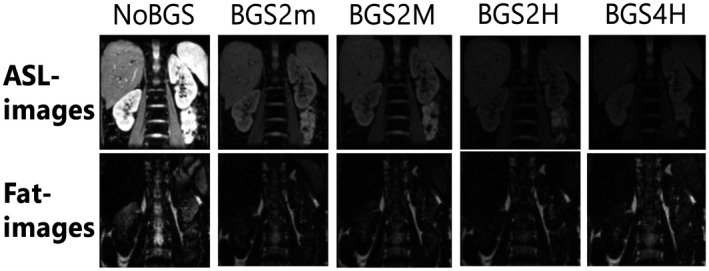
Source images for slice 3 for one subject acquired during free‐breathing in control condition. ASL‐images (top row) for all different BGS levels are shown (columns) with the corresponding fat‐image beneath; for both, the same intensity scaling has been applied. Improvement in fat‐image quality with BGS can be appreciated as superimposed artifacts are reduced. For more details on multislice raw images the reader is referred to Supporting Information Figure S2. ASL, arterial spin labeling; BGS, background suppression

Figure 6 shows raw ASL and fat‐images for one slice acquired in control condition during free‐breathing for different BGS levels. Due to BGS the static tissue signal is extremely reduced. In the Supporting Information Figure S2 an extended version shows all acquired slices, where signal recovery across slices can be observed.

**Figure 7:**
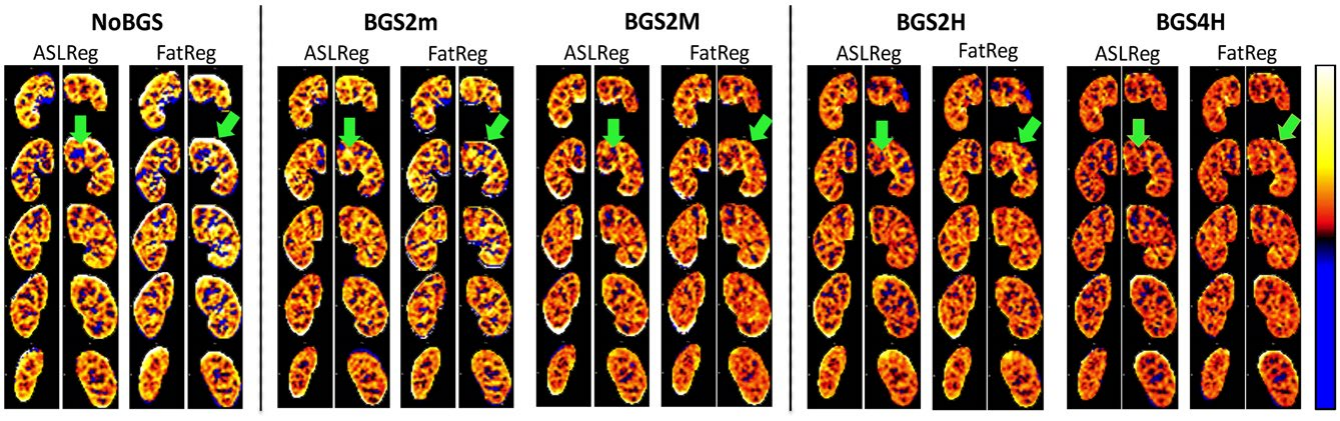
Relative perfusion‐weighted images (PWIs) for multislice pCASL after image registration on ASL‐images (ASLReg) or fat‐images (FatReg). Images are shown for all BGS levels acquired during free‐breathing. Misalignment of control and label images causes subtraction artifacts that appear as extreme erroneous PWS, corrupting the mean PWS inside the kidney ROI. With BGS, artifacts are substantially reduced. Green arrows point out artificially low perfusion inside the kidney and extreme erroneous values around the kidney ROI that are reduced with the application of BGS. ASL, arterial spin labeling; BGS, background suppression; pCASL, pseudocontinuous arterial spin labeling; ROI, region of interest

**Figure 8:**
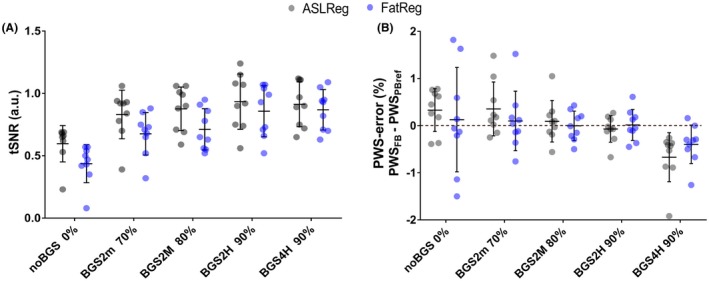
A, Free‐breathing pCASL tSNR for each of the subjects (*n* = 9) with mean and standard deviation. B, Relative perfusion‐weighted signal (PWS) difference to the paced‐breathing reference (dashed line), i.e. PWS‐error [%] [= PWS_FB_ (%) – PWS_PBref_ (%)], with mean and standard deviation bars. Results after ASLReg (gray circles) and FatReg (blue circles) are displayed beside each other for each BGS level. With BGS, an increase in tSNR and a smaller PWS error can be appreciated regardless of the image registration method. However, with the application of four BGS pulses the error is largest. An example for PWIs per repetition, before averaging, illustrating the effect of BGS on tSNR improvement is provided in Figure 9. Exact values per subject (Table S1) as well as the mean and standard deviation at group level (Table S2) can be found in Supporting Information Table S1 and Table S2. ASLReg, arterial spin labeling registration; FatReg, fat registration; pCASL, pseudocontinuous ASL; tSNR, temporal signal‐to‐noise ratio

**Figure 9:**
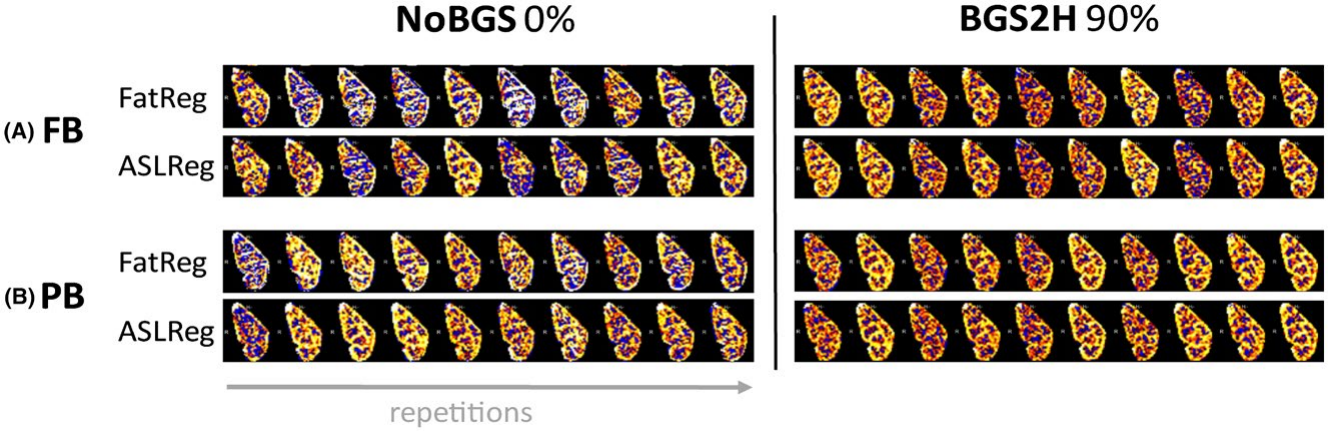
Example of the effect of BGS on temporal SNR increase, i.e. perfusion‐weighted signal (PWS) robustness over time. Displayed are perfusion–weighted images of the left kidney for one subject for a single‐slice per repetition. In the first column without BGS (NoBGS 0% suppression) and in the second column with heavy BGS using two inversion pulses (BGS2H 90% suppression). A, Free‐breathing (FB) results as well as B, paced‐breathing (PB) results are presented after either ASLReg or the proposed FatReg. For both breathing strategies, the PWS is found more robust and less variable with heavy BGS than without. Similarity between results after FatReg and ASLReg on ASL‐images with heavy BGS can be appreciated. ASLReg, arterial spin labeling; BGS, background suppression; FatReg, registration guided by fat‐images; SNR, signal‐to‐noise ratio

With BGS, the PWIs visually improved and showed less extreme values (Figure 7), which was confirmed by an increase in tSNR (Figures 8A, 9) and a reduction in PWS‐error (Figure 8B). The tSNR improvement was significant for all BGS levels compared with NoBGS (*P *< .05), regardless of the registration method. Without BGS tSNR was 0.60 ± 0.15/0.44 ± 0.15 and mean PWS‐error 0.33/0.13% after ASLReg/FatReg, respectively, as compared to a PWS_PBref_ of 2.14 ± 0.79%. With BGS2H tSNR reached 0.93 ± 0.22/0.86 ± 0.20 after ASLReg/FatReg, respectively. A slightly negative trend in PWS was seen with BGS. With BGS2H, the PWS approached the chosen reference, leaving only a small mean PWS‐error of 0.7/0.1% after ASLReg/FatReg, respectively. The largest PWS‐error was observed after ASLReg when four pulses were used for heavy BGS (BGS4H) and only then significant (*P *< .05). For all BGS levels, ASLReg yielded slightly superior tSNR to FatReg (*P *< .05); however, it is noticeable that with advancing BGS level, ASL and FatReg provided similar mean tSNR (Figures 8A, 9).

### 3.3 Part 3: Breathing strategy

**Figure 10:**
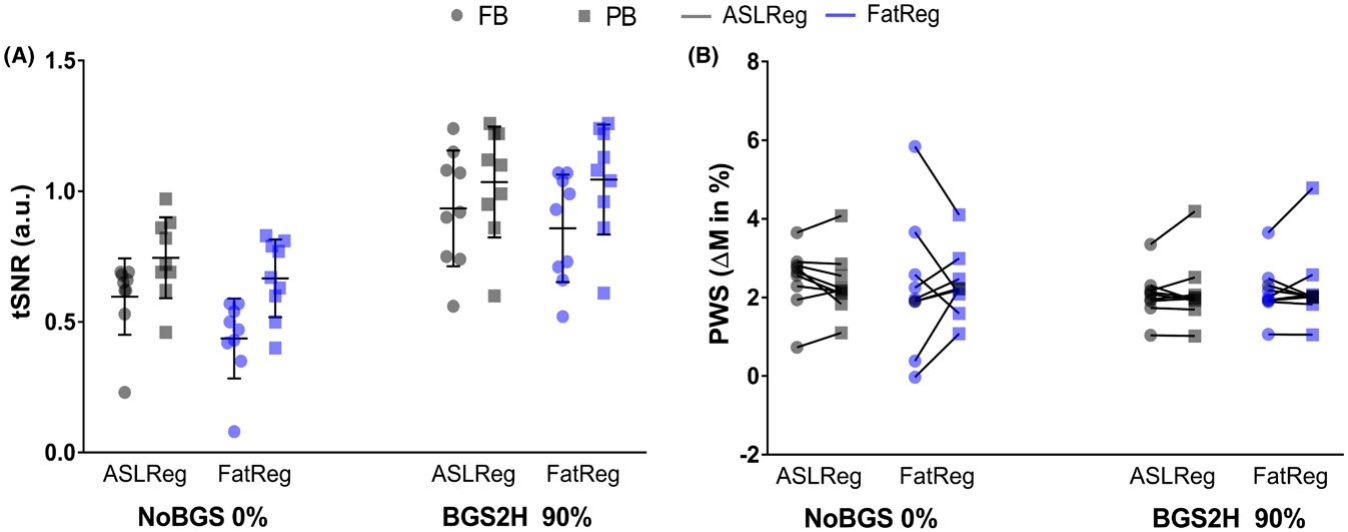
A, Temporal signal‐to‐noise ratio (tSNR) and B, perfusion‐weighted signal (PWS) for each subject. For comparison of free‐breathing (FB; circles) and paced‐breathing (PB; squares), tSNR and PWS are shown without BGS (NoBGS) and for the BGS setting (BGS2H), which was found the most profitable for FB ASL quality in Part 2. Results after ASLReg (grey) and FatReg (blue) are shown beside each other for each scan. With BGS2H the two breathing strategies result in similar tSNR as well as PWS and the PWS spread gets smaller, regardless of the registration technique. In the Supporting Information exact values per subject (Supporting Information Table S1) as well as the mean and standard deviation at group level (Supporting Information Table S2) can be found. ASL, arterial spin labeling; ASLReg, registration guided by ASL‐images; BGS, background suppression; FatReg, registration guided by fat‐images

Overall, visual inspection of raw ASL‐images acquired during free‐breathing and paced‐breathing did not show clear differences (Supporting Information Figure S2, rows 2 and 3). Figure 10 focuses on the free‐breathing and paced‐breathing results for NoBGS and ‐BGS2H; for the other BGS levels the reader is referred to the Supporting Information Figure S3. In general, a tSNR increase was observed for both breathing strategies with BGS2H compared with NoBGS. Using BGS2H, differences in tSNR between free‐breathing and paced‐breathing are reduced, independent of the registration method, with 0.93/0.86 for free‐breathing and 1.04/1.05 for paced‐breathing (for ASLReg/FatReg, respectively). To visualize improved tSNR due to BGS and the subsequent similarity between free‐breathing and paced‐breathing, Figure 9 shows singleslice PWIs before averaging of the label–control subtraction images after both registration methods. Also, the PWS approached similar values for both breathing strategies using BGS2H, with 2.07/2.16% for free‐breathing and 2.14/2.26% for paced‐breathing, leaving only a small difference without significance (*P *> .05). Moreover, the PWS spread decreased and consistency on the level of the individual subjects increased (intrasubject variability for ASLReg/FatReg: NoBGS 0.44/1.23%, BGS2H 0.35/0.48%), for both registration methods.

## 4 DISCUSSION

In this work, we demonstrated the feasibility of free‐breathing renal ASL by combining BGS and retrospective image registration. We found that retrospective image registration of the ASL‐images themselves (ASLReg) still worked for <  90% background suppressed images. Moreover, we demonstrated the potential of additionally acquiring fat‐images in a pCASL sequence for motion correction, which offer a stable contrast for image registration, thus dealing with possible cross‐contrast registration requirements. Finally, we evaluated the effect of BGS and image registration on free‐breathing renal pCASL precision and accuracy, and compared these results with the paced‐breathing strategy. This study consistently showed that BGS considerably increases precision and accuracy regardless of the registration method. Additionally, we achieved good consistency across pCASL acquisitions with different BGS levels, but did not explicitly study reproducibility, which would be best assessed in a patient population with scan–rescan conditions. Subtraction artifacts and noise were reduced and, by that, perfusion maps improved visually as well as quantitatively in terms of tSNR and PWS accuracy. In combination with heavy BGS, free‐breathing renal pCASL showed performance that is on par with the clinically impractical paced‐breathing strategy. Thus, our results demonstrate the potential of free‐breathing renal pCASL, including the combination of BGS and retrospective motion correction, either guided by ASL‐images or guided by fat‐images, as a clinically viable technique for renal perfusion imaging.

In our experience, even with cooperative subjects, paced‐breathing was not always maintained well throughout the entire scan. In fact, we saw that subjects become more conscious of their breathing, evoking sharper and deeper breaths, similar to observations by other groups.^6^, ^8^ This made paced‐breathing data from 1 out of 10 subjects unusable for our analysis, and we encountered hyperventilation in another subject. We anticipate that for patients who experience pain or for children, it might be even more complicated to perform scans with paced‐breathing. By employing free‐breathing as opposed to paced‐breathing TR can be shortened, allowing scan time to be shortened by half or to increase SNR by acquiring more signal averages in the same scan time. This was not investigated in the scope of this study, but is considered for future research. Ultimately, we showed that in combination with BGS, free‐breathing ASL reaches similar quality to paced‐breathing, enabling its usage for clinical applications. Future studies should include patients to verify free‐breathing BGS pCASL feasibility in patients and its sensitivity and specificity to pathology.

We found that, regardless of the registration method, BGS improves ASL quality in terms of tSNR and PWS. While for the brain the positive effect of BGS on ASL quality is well explored and strongly recommended,^14^ its influence on abdominal ASL quality has not been fully investigated as yet and results reported so far are variable.^6^ First reports by De Bazelair et al^7^ state reduction of bulk motion‐induced subtraction artifacts with the application of BGS, which greatly increased the precision of abdominal ASL for clinical imaging, even though breath holding was performed. Likewise, Cutajar et al^5^ used BGS in combination with triggering and, although they reported a decrease in labeling efficiency, acknowledge reduction in noise level and with that improved ASL precision. Our results are in line with these earlier reports and strongly support the use of BGS for free‐breathing renal pCASL. Robson et al^24^ also found that BGS reduces image noise and subtraction artifacts induced by bulk respiratory motion. Nevertheless, free‐breathing abdominal perfusion maps with BGS did have a slightly blurred appearance due to motion‐induced mismatch, implying that BGS was beneficial for ASL quality, but was not sufficient to eliminate image artifacts entirely for free‐breathing ASL by itself.

To visually and quantitatively improve perfusion maps further, retrospective image registration has been found essential.^6^, ^10^, ^25^ Interestingly, negative results on SNR, reproducibility, and measured perfusion rate are reported for the combination of image registration and BGS.^6^ Previous reports indicated that image registration is complicated by the lack of static tissue contrast due to BGS^15^, ^16^; however, this issue has not been experimentally investigated as yet. In the current study, we did not find reduced ASL quality after image registration on free‐breathing BGS pCASL‐images but instead obtained the best ASL quality with this setting. Those findings are supported by recent work of Taso et al., who corrected for motion in free‐breathing renal pCASL by 2D image registration on a BGS singleslice ASL‐image. Here, we increased the kidney coverage by using multislice acquisition, employed 3D registration, and introduced an additional approach to correct for motion using fat‐images, which could serve to overcome potential registration issues of BGS images by providing a stable contrast that is unaffected by BGS. Similarly, in a recent report, Nery et al., demonstrated tSNR improvement after 3D rigid image registration for background‐suppressed 3D GRASE FAIR ASL; however, no comparison was made with non‐BGS data. Furthermore, we observed a reduced spread in PWS between subjects with increased levels of BGS. This reduction in signal spread was reported previously and was interpreted to offer greater sensitivity for ASL.^11^ Besides, we found a small decrease in PWS, related to the number of inversion pulses applied, in line with previous reports on inversion efficiency of hyperbolic secant inversion pulses and its effect on the ASL signal.^11^, ^14^, ^26^ We validated the inversion efficiency of the utilized BGS inversion pulse in a phantom experiment and found an efficiency of 0.98. Since we did not find a benefit in tSNR using more than two inversion pulses, but did observe a decrease in PWS with four inversions, it might be advisable to keep the number of inversion pulses low. Still, even with a small signal loss, BGS free‐breathing PWS approached the reference and the gain in precision as well as accuracy realized by BGS is substantial. In addition, BGS had a positive effect on fat‐image quality, as superimposed water‐signal artifacts in the fat‐images were reduced (Figure 6), leading to improved FatReg performance on par with conventional ASLReg.

In the scope of this study BGS inversion pulse timings have been implemented to suppress kidney tissue signal to the desired level for the first slice. Due to the multislice character of our sequence, BGS across slices is inherently inhomogeneous, making extensive optimization for the purpose of this work seem superfluous. However, suggestions for improving BGS for multislice acquisitions have recently been made.^12^, ^26^

When comparing motion correction using ASL‐images with different BGS levels, even for 90% BGS, ASLReg yielded valid image registration results for our multislice gradient echo with echo planar imaging readout and the utilized registration algorithm despite minimal structural image contrast in BGS ASL‐images (Figure 6 and Supporting Information Figure S2). On the other hand, initial findings indicated issues using ASLReg for the M0 coregistration to the BGS ASL data, due to different image contrasts, which was also reported previously.^8^ Therefore, we chose to perform the M0 coregistration based on fat‐images. For reference, we investigated the performance of the BGS ASL‐images themselves for that M0 coregistration, which had a success rate of 54% as opposed to 100% for FatReg. Note that in the scope of this study we did not optimize the registration for cross‐contrast problems; nor did we investigate the application of other registration methods. Clearly, the poor coregistration, with frequent erroneous translations of ASLReg, makes the results invalid for further analysis (Supporting Information Figure S4). Despite varying fat‐contour quantity among subjects (Supporting Information Figure S5), FatReg offered the potential for M0 coregistration for all BGS levels, as was already performed in this study.

A limitation of our proposed fat‐image approach is that the delay between the ASL‐image and fat‐image acquisition gets longer with the number of slices, increasing the risk of motion and thus mismatch during the delay. Ideally, to acquire a fat‐image that is most representative for the motion state of the corresponding ASL‐image, acquisitions are desired to be as close as possible. Motion between the corresponding images would result in improperly aligned ASL‐images with subtraction artifacts in the PWIs as a consequence. As we found that FatReg performance significantly decreases for delays longer than 400 ms, the number of acquired slices should be chosen carefully, so they do not lead to longer delays. We observed that five slices as acquired in this study sufficiently cover the kidneys and appreciate the feasibility of the FatReg technique for multislice renal ASL, as opposed to singleslice ASL. For our acquisition with five slices, the delay between the corresponding ASL‐image and fat‐image was 313 ms. Respiratory‐induced kidney motion has been reported in the range of 2 to 4 cm^27^, ^28^; with a breathing cycle duration of 2 to 5 s, a speed of 0.4 to 2 cm/s can be expected during respiration. On top of that, subjects most often fall into a slow, shallow breathing pattern during free‐breathing acquisitions.^8^ Thus, kidney displacement is expected to be limited during the 313‐ms delay between the corresponding ASL and fat‐image. Still, FatReg is slightly outperformed by the standard ASLReg for motion between BGS ASL‐images; that could indicate motion during the delay and, consequently, a small residual misalignment after FatReg negatively affecting ASL quality. Finally, the additional fat‐image acquisition has a slight scan time increase (65 ms per slice); however, on a typical ASL acquisition it would mean a 14% increase for a five‐slice pCASL acquisition including fat‐images.

For clinical feasibility, it would be helpful to automate postprocessing further by eliminating the manual input for setting the kidney ROI used for registration and quantification. In this, machine learning offers potential but the specific contrast differences in the ASL‐images due to varying BGS levels would call for dedicated methodology and thus need further investigation.

Our results demonstrate that motion correction between BGS ASL‐images using ASLReg is feasible, even for heavily suppressed images up to 90%, but might depend on the specific readout and image registration algorithm. The FatReg is expected to offer the possibility of registering ASL‐images with even stronger background suppression. In the scope of this study, however, we did not investigate BGS stronger than 90% to prevent negative signals that would require analysis using complex or phased data. Because of phase effects in our gradient echo readout we were restricted to work with magnitude data.

## 5 CONCLUSION

We found background suppression highly beneficial for free‐breathing renal pCASL, increasing precision without compromising accuracy in combination with either the conventional registration on the ASL‐images themselves or the proposed method based upon guidance by fat‐images. In addition, fat imaging offers a common contrast for cross‐contrast registration problems such as coregistration of the M0 to background suppressed ASL‐images. Furthermore, with the application of heavy background suppression and image registration, free‐breathing renal pCASL has shown similar performance to the reference paced‐breathing strategy, promising a clinically viable method for noncontrast renal perfusion imaging.

## Supporting information


**FIGURE S1** Perfusion‐weighted images (PWIs) for acquisitions with different BGS acquired in free‐breathing (FB, top) and paced‐breathing (PB, bottom) for subject 2, who had major PB issues during two scans. Because of extensive through‐plane motion during PB the kidney shape changed so that the registration algorithm could not align the images properly, resulting in PWS maps with corrupting subtraction artifacts. BGS, background suppression
**FIGURE S2** Source images for all five slices (horizontal) for one subject. The equilibrium magnetization image M0 in the top row, followed by ASL‐images for all different BGS levels. Signal recovery across the slices is apparent. In rows 2 and 4 raw control (C) and label (L) images of one label–control pair are illustrated for a scan without BGS (NoBGS). All images were acquired in free‐breathing (FB). Except for row 3, displaying a control image acquired during paced‐breathing (PB). Visually, raw ASL‐image quality is similar for FB and PB scans. In the last two rows, fat‐images without BGS (NoBGS) and for BGS4H are shown. Improvement in fat‐image quality with BGS can be appreciated as superimposed artifacts are reduced. ASL, arterial spin labeling; BGS, background suppression
**FIGURE S3** A, Temporal signal‐to‐noise ratio (tSNR) and B, perfusion‐weighted signal (PWS) of nine included subjects for pseudocontinuous arterial spin labeling (pCASL)acquisitions with different background suppression (BGS) levels. The tSNR and PWS were calculated for all BGS scans after motion correction directly on the ASL‐images (ASLReg, gray) and the fat‐images (FatReg, blue). Results for free‐breathing (FB) scans displayed with circles and for paced‐breathing (PB) scans with squares beside each other. ASLReg, arterial spin labeling registration; FatReg, fat registration
**FIGURE S4** Example of invalid registration results: M0 coregistration of the right kidney for subject 9 (slices 1‐5, rows) to heavily background suppressed scan (BGS4H), guided by either ASLReg or FatReg. M0‐image, background suppressed ASL‐images, as well as fat‐images are shown. The red‐dotted line indicates the target position of the kidney bottom in the suppressed ASL‐images in slice 5. The red arrow is pointing at the bottom of the unregistered kidney in the M0. Erroneous translation of the M0 is observed after ASLReg is due to reduced image contrast. In contrast, FatReg resulted in valid alignment, as shown by the green arrow pointing at the bottom of the kidney after FatReg, which is now in line with the line. ASLReg, arterial spin labeling registration; FatReg, fat registration
**FIGURE S5** Fat contours for five slices (rows) of all acquired subjects (columns). Variation in fat quantity among subjects is present as well as fat‐signal recovery along slices
**TABLE S1** Temporal signal‐to‐noise ratio (tSNR) and perfusion‐weighted signal (PWS) for all acquired subjects for pCASL acquisitions with different BGS levels (suppression levels in percentages). The tSNR and PWS were calculated for all BGS scans after motion correction on the ASL‐images themselves (ASLReg) and on the fat‐images (FatReg). Rows 1‐5 contain results for free‐breathing (FB) scans and rows 6‐10 for paced‐breathing (PB) scans. Corrupted result for subject 2 is displayed in gray as it has been excluded from the analysis. ASLReg, arterial spin labeling registration; BGS, background suppression; FatReg, fat registration; pCASL, pseudocontinuous ASL
**TABLE S2** Mean (standard deviation) for temporal signal‐to‐noise ratio (tSNR) and perfusion‐weighted signal (PWS) including nine subjects for pCASL acquisitions with different BGS levels (suppression levels in %); tSNR and PWS were calculated for all BGS scans after motion correction on the ASL‐images themselves (ASLReg) and on the fat‐images (FatReg). In the two main columns results for scans acquired during free‐breathing (FB) and paced‐breathing (PB) are shown. For FB, additionally the PWS error as comparison to the chosen reference value is presented. ASLReg, arterial spin labeling registration; FatReg, fat registration; BGS, background suppression; pCASL, pseudocontinuous ASL
