# Supplementary material for: Enabling free‐breathing background suppressed renal pCASL using fat imaging and retrospective motion correction
Source: Magn Reson Med. 2019 Mar 18;82(1):276–88. doi: 10.1002/mrm.27723 (PMC6593735; doi:10.1002/mrm.27723)
Supplement: Supplementary file 1 — FIGURE S1 Perfusion‐weighted images (PWIs) for acquisitions with different BGS acquired in free‐breathing (FB, top) and paced‐breathing (PB, bottom) for subject 2, who had major PB issues during two scans. Because of extensive through‐plane motion during PB the kidney shape changed so that the registration algorithm could not align the images properly, resulting in PWS maps with corrupting subtraction artifacts. BGS, background suppression FIGURE S2 Source images for all five slices (horizontal) for one subject. The equilibrium magnetization image M0 in the top row, followed by ASL‐images for all different BGS levels. Signal recovery across the slices is apparent. In rows 2 and 4 raw control (C) and label (L) images of one label–control pair are illustrated for a scan without BGS (NoBGS). All images were acquired in free‐breathing (FB). Except for row 3, displaying a control image acquired during paced‐breathing (PB). Visually, raw ASL‐image quality is similar for FB and PB scans. In the last two rows, fat‐images without BGS (NoBGS) and for BGS4H are shown. Improvement in fat‐image quality with BGS can be appreciated as superimposed artifacts are reduced. ASL, arterial spin labeling; BGS, background suppression FIGURE S3 A, Temporal signal‐to‐noise ratio (tSNR) and B, perfusion‐weighted signal (PWS) of nine included subjects for pseudocontinuous arterial spin labeling (pCASL)acquisitions with different background suppression (BGS) levels. The tSNR and PWS were calculated for all BGS scans after motion correction directly on the ASL‐images (ASLReg, gray) and the fat‐images (FatReg, blue). Results for free‐breathing (FB) scans displayed with circles and for paced‐breathing (PB) scans with squares beside each other. ASLReg, arterial spin labeling registration; FatReg, fat registration FIGURE S4 Example of invalid registration results: M0 coregistration of the right kidney for subject 9 (slices 1‐5, rows) to heavily background suppressed scan (BGS4H), guided by either A [file MRM-82-276-s001.docx]

Figure S1: Perfusion weighted images (PWIs) for acquisitions with different BGS acquired in free-breathing (FB, top) and paced-breathing (PB, bottom) for subject 2 that had major PB issues during two scans. Due to extensive through-plane motion during PB the kidney shape changed so that the registration algorithm couldn’t align the images properly, resulting in PWS maps with corrupting subtraction artifacts.

Figure S2: Source images for all 5 slices (horizontal) for one subject. The equilibrium magnetization image M0 in the top row, followed by ASL-images for all different BGS-levels. Signal recovery across the slices is apparent. In rows 2 and 4 raw control (C) and label (L) images of one label-control pair are illustrated for a scan without BGS (NoBGS). All images were acquired in free-breathing (FB). Except for row 3, displaying a control image acquired during paced-breathing (PB). Visually, raw ASL-image quality is similar for FB and PB scans. In the last two rows, fat-images without BGS (NoBGS) and for BGS4H are shown. Improvement in fat-image quality with BGS can be appreciated as superimposed artifacts are reduced.

Figure S3: (a) Temporal SNR (tSNR) and (b) perfusion weighted signal (PWS) of 9 included subjects for pCASL acquisitions with different BGS-levels. tSNR and PWS were calculated for all BGS scans after motion correction directly on the ASL-images (ASLReg, grey) and the fat-images (FatReg, blue). Results for free-breathing (FB) scans displayed with circles and for paced-breathing (PB) scans with squares next to each other.

Figure S4: Example for invalid registration results: M0 co-registration of the right kidney for subject 9 (slice 1-5, rows) to heavily background suppressed scan (BGS4H), guided by either ASLReg or FatReg. M0-image, background suppressed ASL-images as well as Fat-images are shown. The red-dotted line indicating the target position of the kidney bottom in the suppressed ASL-images in slice 5. The red arrow is pointing at the bottom of the unregistered kidney in the M0. Erroneous translation of the M0 is observed after ASLReg due to reduced image contrast. In contrast, FatReg resulted in valid alignment, as shown by the green arrow pointing at the bottom of the kidney after FatReg, which is now in line with the line.

Figure S5: Fat-contours for 5 slices (rows) of all acquired subjects (columns). Variation in fat-quantity among subjects is present as well as fat-signal recovery along slices.

Table S1: Temporal SNR (tSNR) and perfusion weighted signal (PWS) for all acquired subjects for pCASL acquisitions with different BGS-levels (suppression levels in %). tSNR and PWS were calculated for all BGS scans after motion correction on the ASL-images themselves (ASLReg) and on the fat-images (FatReg). Row 1-5 containing results for free-breathing (FB) scans and row 6-10 for paced-breathing (PB) scans. Corrupted results for subject 2 displayed in grey as it has been excluded from the analysis.

Table S2: Mean (standard deviation) for temporal signal-to-noise ratio (tSNR) and perfusion-weighted signal (PWS) including 9 subjects for pCASL acquisitions with different BGS-levels (suppression levels in %); tSNR and PWS were calculated for all BGS scans after motion correction on the ASL-images themselves (ASLReg) and on the fat-images (FatReg). In the two main columns results for scans acquired during free-breathing (FB) and paced-breathing (PB) are shown. For FB, additionally the PWS-error as comparison to the chosen reference value is presented.
